# Social comfort and attractiveness perception: impact of prosthetics, physical disability and comfort distance on interpersonal interactions

**DOI:** 10.1017/ehs.2025.5

**Published:** 2025-02-14

**Authors:** Farid Pazhoohi, Samantha Wing, Alan Kingstone

**Affiliations:** 1 School of Psychology, University of Plymouth, Plymouth, UK; 2 McGill Cognitive Science Program, McGill University, Montreal, QC, Canada; 3 Department of Psychology, University of British Columbia, Vancouver, BC, Canada

**Keywords:** physical disability, comfort distance, attractiveness, pathogen disgust, perceived vulnerability to disease

## Abstract

Derived from the disease-avoidance model is the hypothesis that people may direct negative cognitive and behavioural responses towards individuals with physical disfiguring conditions, including physical disabilities. According to the behavioral immune system, physical disability—a non-contagious physical disfigurement—may falsely activate cognitive disease-avoidance processes, resulting in prejudicial or negative responses toward individuals with physical disabilities. For the first time this hypothesis is put to the test by investigating whether ratings of attractiveness and comfort for a social interaction vary systematically with physical disability (Studies 1 and 2). In addition, we tested whether these ratings were associated with individual differences in pathogen disgust and perceived vulnerability to disease. In Study 3 we overcame possible methodological limitations by employing a virtual reality (VR) environment. A fourth study was conducted to extend the first two studies by using a more diverse set of avatars. Results from Studies 1 and 2 indicated that disability did not significantly impact comfort ratings for social interactions, although non-disabled stimuli were rated as more attractive. However, Study 3 showed that in a VR environment, participants preferred closer proximity to non-disabled avatars over disabled ones, a preference not mitigated by the presence of prosthetics. Study 4 replicated these findings with varied 2D avatars, showing that disability significantly affected both comfort and attractiveness ratings, with non-disabled avatars rated highest, followed by those with prosthetics, and finally disabled avatars. Despite these findings, the expected relationship between comfort ratings and individual differences in pathogen disgust or perceived infectability did not emerge, challenging the behavioural immune system proposal. The discomfort associated with physical disability may be more related to social stigma or preconceived notions than to an innate disease-avoidance response.

## Graphical abstract

**Figure:**
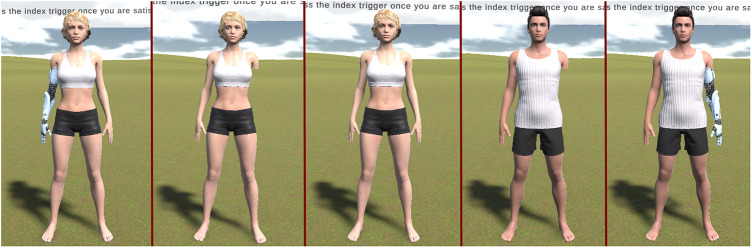

## Social Media Summary

Does physical disability activate disease-avoidance biases? Our studies reveal mixed findings, challenging the behavioural immune system.

## 1. Introduction

Two decades ago, in a theoretical paper, Park, Faulkner, and Schaller (2003) proposed that an extension of the disease-avoidance model suggests a behavioural immune system whereby negative cognitive and/or emotional reactions may be elicited towards those with physical disabilities and other physically disfiguring conditions. This in turn could lead to prejudicial attitudes and avoidance behaviours towards individuals with physical disabilities and disfiguring conditions. This proposition of the behavioural immune system (Murray & Schaller, 2016) holds that humans evolved psychological mechanisms to avoid interactions and contacts with those who are perceived as carrying communicable pathogens and diseases (Kurzban & Leary, 2001; Thornhill & Fincher, 2014). According to Park et al. (2003), the non-contagious physical disfigurations may activate cognitive disease-avoidance processes, which act as ‘false positives’ (i.e. incorrectly tagging a healthy individual as diseased). This in turn can result in prejudicial or negative responses against individuals with physical disabilities including physical avoidance and/or elevated levels of anxiety (Murray & Schaller, 2016; Oaten, Stevenson, & Case, 2011; Park et al., 2003).

This hypothesis was recently put to the test by Pazhoohi and colleagues (2022), who presented participants with images of physically disabled and non-disabled individuals and asked the observers of the opposite sex to rate how attractive they found each individual as a romantic partner. Participants then completed disgust (Tybur, Lieberman, & Griskevicius, 2009) and disease scales (Duncan, Schaller, & Park, 2009) to test for how their ratings of attractiveness may have been linked to these measures. The findings failed to support the predictions of the behavioural immune system. Not only was no association between romantic partner preference and the scales found, but women reported greater preference for physically disabled men than non-disabled men. This latter finding has been replicated in another study in which women rated disabled men as generally more attractive than non-disabled men (Pazhoohi, Capozzi, & Kingstone, 2021). It can be argued that the concept of attractiveness, which is inherently based on a positive perception, may be biased against the hypothesis proposed by the behavioural immune system and its suggestion that people tend to avoid physical contact with individuals who have disabilities (Park et al., 2003). The current research investigated this possibility.

Comfort distance provides a reliable tool for measuring implicit and explicit attitudes toward social interactions and situations (Hall, 1966; Sundstrom & Altman, 1976). Comfort distance increases in uncomfortable and threatening situations and decreases in non-threatening and comfortable situations (Iachini et al., 2016; Kramer et al., 2020; Pazhoohi et al., 2019; Taffou & Viaud-Delmon, 2014). We used comfort distance as a measure in the present research, with the prediction being that individuals should prefer a larger distance from disabled individuals relative to non-disabled individuals. Accordingly, in the first study we recruited a sample of self-identified non-disabled individuals and asked them to indicate the degree to which they felt comfortable interacting with a 2D female model who was presented at different distances from the observer. The female model varied in terms of being non-disabled or wearing a prosthetic. The aim of this study was to investigate whether wearing a prosthetic enhances comfort and physical attractiveness ratings to a level comparable to individuals without disabilities. Although some researchers have speculated on the positive behavioural and perceptual impact of prosthetic limbs (e.g., Murray & Fox, 2002; Tamari, 2017), the issue remains to be addressed empirically. To further explore the effect of physical disability, in the second study, another sample of participants was recruited and using a design similar to Study 1, they were presented with female non-disabled models or models with a disability (i.e., an amputated arm). Moreover, in both the studies, the results were examined with regard to individual difference measures of pathogen disgust (Tybur et al., 2009), perceived vulnerability to diseases (Duncan et al., 2009), and concerns about contracting COVID-19, predicting that comfort ratings for disabled models will be negatively associated with these individual differences. Study 3 addressed possible limitations of Studies 1 and 2 by using a virtual reality (VR) environment, including both male and female models, and instead of comfort ratings, it specifically measured the comfort distance for a social interaction. A fourth study was conducted to extend the first two studies by using a more diverse set of avatars.

## 2. Study 1

The objective of the first study was to explore if wearing prosthetics enhances comfort and physical attractiveness ratings in a manner comparable to individuals without disabilities.

### 2.1. Method

#### Participants

A total of 120 individuals (48 men and 72 women), aged between 19 and 78 years (*M* = 39.2, *SD* = 13.5), were recruited from CloudResearch during the COVID-19 pandemic in the winter of 2021. A total of 56 participants (46.7%) reported being married, and 15.0% reported being not married but in a relationship. Additionally, 30.0% reported being single, and 8.3% were either widowed, divorced or separated. As for their highest educational degree, 24.2% had a high school diploma, 5.8% had a post-secondary diploma, 51.7% had an undergraduate degree and 18.3% had a postgraduate degree. Informed consent was obtained from all subjects involved in all the studies of this research. This and the next studies were conducted according to the guidelines of the Declaration of Helsinki and approved by the Behavioural Research Ethics Committee of the University of British Columbia.

#### Measures

*Perceived vulnerability to disease*: The 15-item Perceived Vulnerability to Disease self-report instrument (Duncan et al., 2009) was used to measure individuals’ chronic concerns about the transmission of infectious diseases. The answers were on a 7-point Likert scale from 1 (*strongly disagree*) to 7 (*strongly agree*), with higher values indicating higher perception of vulnerability to diseases. The scale is composed of two subscales of *perceived infectability*, which assesses beliefs about one’s own susceptibility to infectious diseases, and *germ aversion*, which assesses emotional discomfort in contexts that connote high potential for pathogen transmission (Duncan et al., 2009).

*Pathogen disgust:* A seven-item pathogen disgust scale from Three Domains of Disgust Scale (Tybur et al., 2009) was used to measure individual differences in pathogen disgust. The answers range from 0 indicating *not at all disgusting* to 6 indicating *extremely disgusting* (Tybur et al., 2009). The study did not include the two additional subscales of sexual and moral disgust, and consequently no data were collected for these dimensions.

*COVID-19:* We asked participants to provide their answers about their attitude towards COVID-19 disease on a 7-point Likert scale from *very low* (1) to *very high* (7) for the following questions: ‘How concerned are you in general about the coronavirus outbreak?’ and ‘When you are in public how concerned are you about contracting the coronavirus?’.

#### Stimuli and procedure

**Figure 1.**
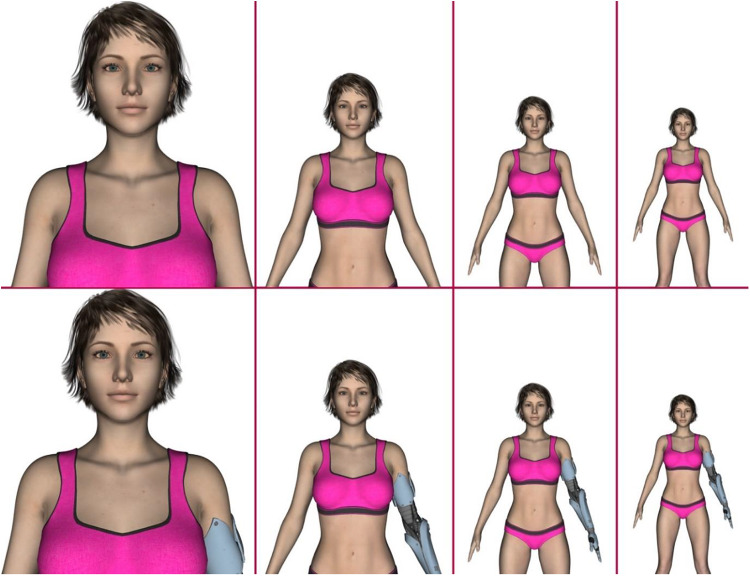
Stimuli in distances of 100, 200, 300 and 400 cm from camera from left to right for body-abled (upper row) and prosthetics (lower row) blocks.

A Caucasian female avatar was implemented using Daz3d software (www.daz3d.com). The avatar was positioned forward facing in front of the camera (see Fig. 1). The distance varied from 100 cm to 400 cm away from the camera, with increments of 10 cm, resulting in 31 different stimuli. To create the prosthetic condition, another set of 31 stimuli of the same images were created by adding a prosthetic arm replacing the left arm of the avatar.

At the beginning of the study participants completed a series of questionnaires (demographic, pathogen disgust, perceived vulnerability to disease scale, and COVID-19 questions). Each set of stimuli (non-disabled or with prosthetic) were then presented randomly in separate blocks. As this was a within-subjects design, non-disabled and prosthetic stimulus sets were counter-balanced across participants. For each display participants were asked to respond to the question ‘How comfortable are you interacting with this person at this distance?’ and ‘How attractive do you find this person?’ on a 7-point scale, from 1 (*not at all*) to 7 (*very*).

### 2.2. Results

**Figure 2.**
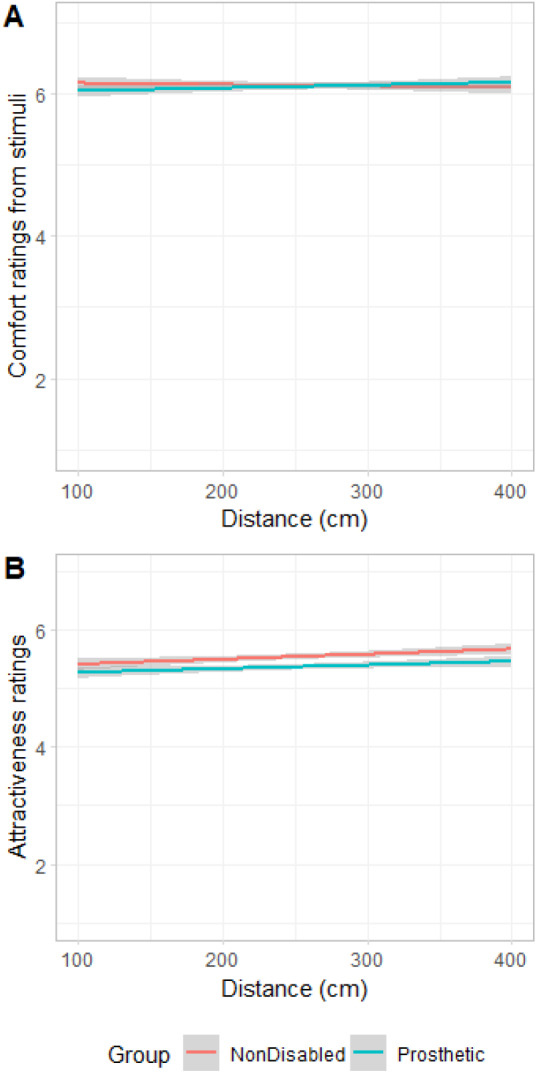
(A) Comfort ratings and (B) attractiveness ratings as a function of distance, and stimuli group (nondisabled and with prosthetics).

A linear mixed model was conducted to investigate the effect of disability, distance and participants’ sex on the comfort ratings for an interaction, with participants as a random factor. Disability and participants’ sex were categorical variables and distance was continuous. Pathogen disgust, germ aversion, perceived vulnerability and concern about contracting COVID-19 were added as covariates to the model. None of the main effects of Disability and Participants’ Sex, Distance, and their interactions were significant (see Table 1 for details of the model; Fig. 2A). Moreover, none of the covariates showed any significance (Table 1). All post-hoc comparisons reported here, and throughout the results, were done using Bonferroni correction, and this is reflected in the *p* values.

**Table 1.** Estimates for the effects of distance, group (disability vs. prosthetic) and participant sex, as well as pathogen disgust, germ aversion, perceived infectability and concern about contracting COVID-19 on comfort and attractiveness ratings

	Comfort ratings					Attractiveness ratings				
	Estimate	*SE*	*df*	*t*	*p*	Estimate	*SE*	*df*	*t*	*p*
(Intercept)	6.39	1.09	121	5.88	<.001^***^	4.04	1.30	120	3.09	.002**
Distance	−0.01	0.01	7320	−1.04	.300	0.01	0.01	7320	1.97	.049*
Group (prosthetic)	−0.12	0.09	7320	−1.34	.181	−0.04	0.07	7320	−0.61	.539
Sex (male)	0.17	0.23	155	0.76	.449	0.48	0.26	135	1.81	.072
Distance × group (prosthetic)	0.01	0.01	7320	1.93	.053	−0.01	0.01	7320	−0.73	.465
Distance × sex (male)	0.01	0.01	7320	0.15	.883	0.01	0.01	7320	3.41	<.001***
Group (prosthetic) × sex (male)	−0.09	0.11	7320	−0.80	.423	−0.12	0.09	7320	−1.37	.172
Distance × group (prosthetic) × sex (male)	−0.01	0.00	7320	−0.07	.941	−0.01	0.00	7320	−0.18	.859
Pathogen disgust	0.10	0.11	120	0.95	.367	0.24	0.14	120	1.72	.087
Germ aversion	−0.16	0.12	120	−1.45	.147	−0.01	0.13	120	−0.04	.962
Perceived infectability	−0.01	0.08	120	−0.05	.957	−0.01	0.10	120	−0.04	.964
Concern about contracting COVID-19	−0.02	0.05	120	−0.52	.602	−0.13	0.06	120	−2.05	.041*

*Note:*

* *p* < .05, **p < .01, ***p < .001.

**Figure 3.**
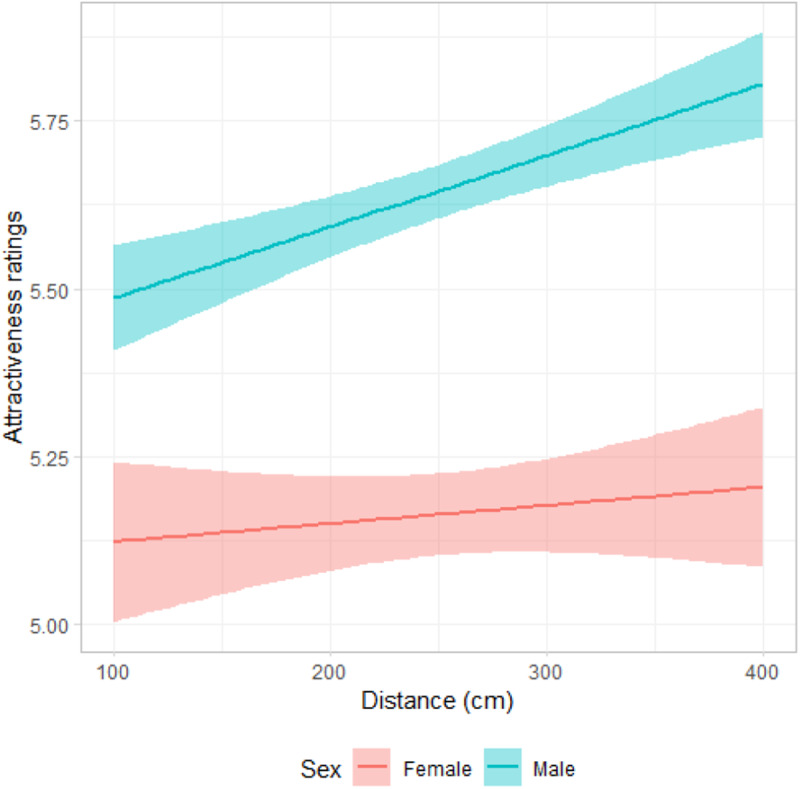
Attractiveness ratings as a function of distance, and participant sex.

Results of the linear mixed model for the attractiveness did not show a significant main effect for disability (non-disabled vs. prosthetics, see Table 1 for details of model), indicating that participants rated stimuli wearing prosthetics similar as non-disabled ones for attractiveness. However, results showed a very small but positive association between distance and attractiveness ratings (*β* = 0.01, *SE* = 0.01, *df* = 7320, *t* = 1.97, *p* = .049; Fig. 2B). In addition, distance and participants’ sex interaction was significant, *β* = 0.01, *SE* = 0.01, *df* = 7320, *t* = 3.41, *p* < .001. Post-hoc analysis showed that men (*M* = 5.66, *SEM* = 0.15, 95% CI [5.35, 6.96]) rated the images more attractive than women (*M* = 5.15, *SEM* = 0.19, 95% CI [4.77, 5.52], *p* = .040; Fig. 3). Moreover, Fig. 3 shows that the participant sex × distance interaction was associated with the attractiveness ratings, reflecting that men’s attractiveness ratings with distance had a higher slope than women’s. In other words, attractiveness increased more drastically with distance for men compared to women. Concern about contracting COVID-19 was negatively associated with attractiveness ratings (Table 1). No other covariates were associated with attractiveness ratings.

### 2.3. Discussion

Results from Study 1 indicated that both men and women preferred a similar distance when presented with images of a non-disabled person and a person with a prosthetic, and both types of images were considered equally attractive, although overall, men compared to women rated the stimuli more attractive. Furthermore, the finding that attractiveness ratings increased more significantly with distance for men compared to women suggests that as more bodily areas of the female model became visible—such as the breasts, waist, and hips—men’s attractiveness ratings increased. This finding might imply that in the assessment of attractiveness, women placed greater emphasis on other aspects of the model, such as facial features, rather than bodily areas.

The association between comfort ratings and perceived infectability, germ aversion, pathogen disgust, and concern about contracting COVID-19 failed to support the predictions of the behavioural immune system (Park et al., 2003). In contrast to this finding, however, the rating of the attractiveness was negatively associated with concern about contracting COVID-19, suggesting those individuals that were concerned about contracting the virus rated the stimuli overall as less attractive.

## 3. Study 2

Study 1 pursued the question of whether wearing prosthetics can influence ratings of social comfort and attractiveness as a function of distance. The second study used disabled stimuli instead of stimuli wearing prosthetics. In all other respects, the design was the same as in Study 1.

### 3.1. Method

#### Participants

A total of 122 individuals (62 men and 60 women), aged between 20 and 73 years (*M* = 39.8, *SD* = 13.4), were recruited from CloudResearch during the COVID-19 pandemic in the winter of 2021. A total of 42 participants (34.4%) reported being married, and 18.0% reported being not married but in a relationship. Additionally, 36.1% reported being single, and 11.5% were either widowed, divorced or separated. As for their highest educational degree, 26.2% had a high school diploma, 6.6% had a post-secondary diploma, 45.9% had an undergraduate degree and 21.3% had a postgraduate degree.

#### Measures, stimuli and procedure

**Figure 4.**
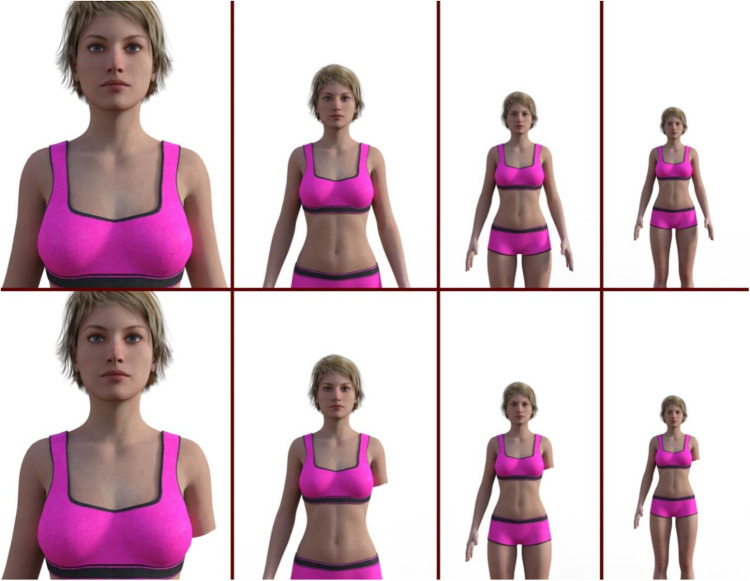
Stimuli in distances of 100, 200, 300 and 400 cm from camera from left to right for body-abled (upper row) and disabled (lower row) blocks.

The measures, stimuli and procedure were equivalent to Study 1, except that a prosthetic was not present for one set of images (Fig. 4). To create this disability condition, the left arm of the non-disabled avatar was removed. Participant instructions referred to this as an amputation. The stimuli in the second study were generated with higher resolution and improved rendering quality.

### 3.2. Results

**Figure 5.**
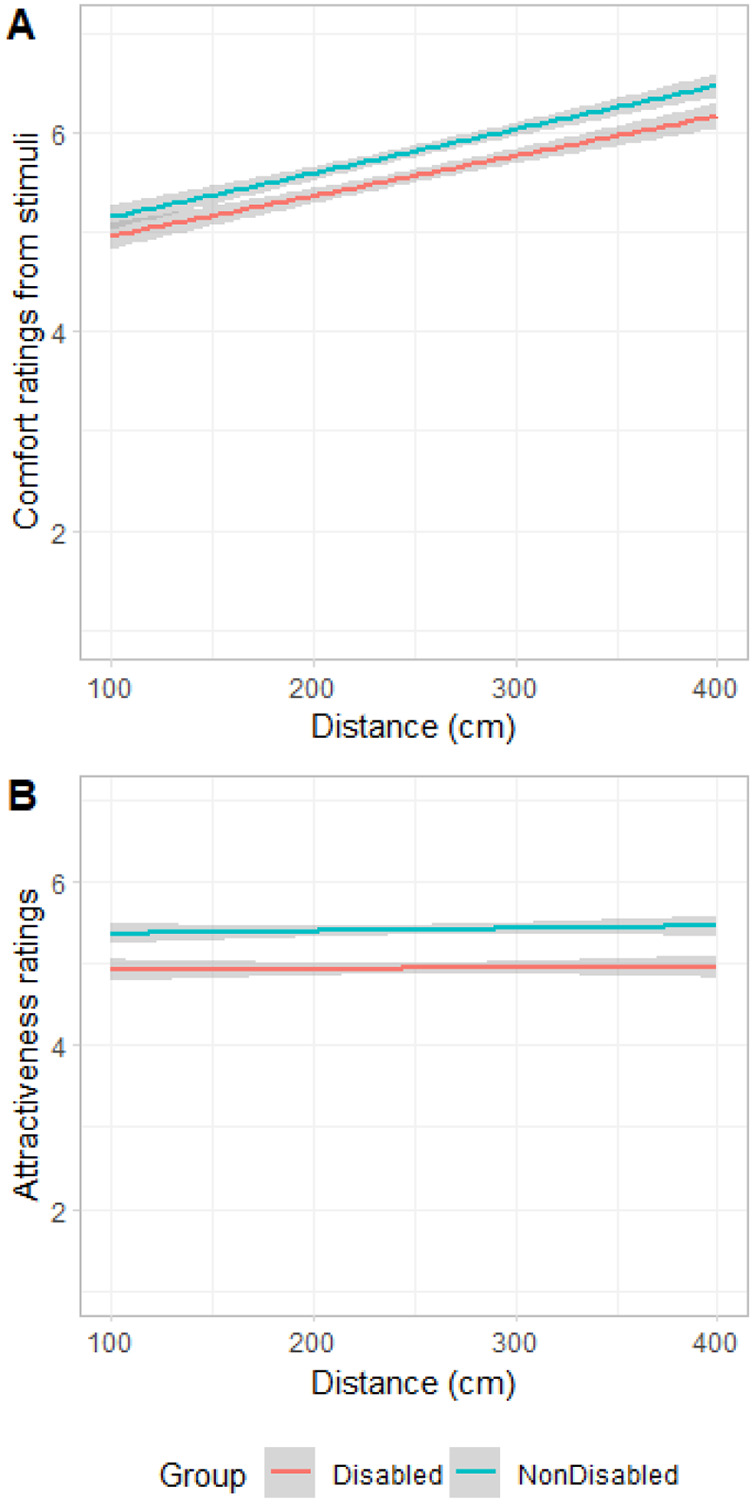
(A) Comfort ratings and (B) attractiveness ratings as a function of distance, and stimuli group (disabled and nondisabled).

**Figure 6.**
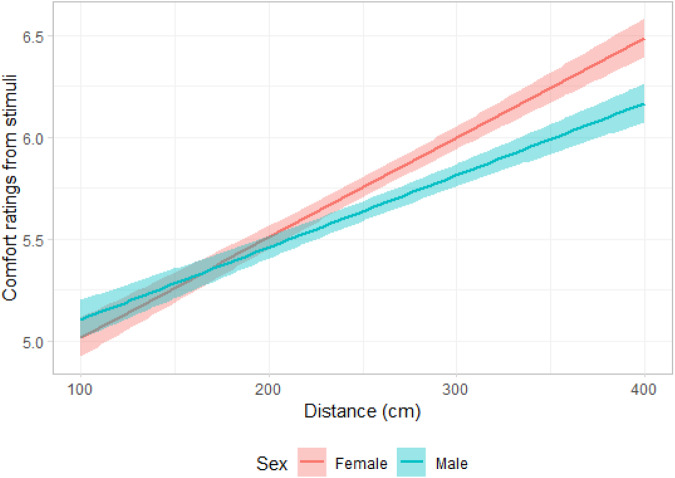
Comfort ratings as a function of distance, and participant sex.

A linear mixed model was conducted to investigate the effect of disability, distance and participants’ sex on the comfort ratings for an interaction, with participants as a random factor. Disability and participants’ sex were categorical variables and distance was continuous. Pathogen disgust, germ aversion, perceived vulnerability and concern about contracting COVID-19 were added as covariates to the model. As illustrated in Fig. 5A, distance was positively associated with comfort ratings, *β* = 0.01, *SE* = 0.01, *df* = 7442, *t* = 19.15, *p* < .001. Figure 6 shows that the participant sex × distance interaction was associated with the comfort ratings, reflecting that men’s comfort ratings with distance had a lower slope than women’s (see Table 2 for details of the model). In other words, comfort increased more drastically with distance for women compared to men. No other variable was associated with the comfort ratings (Table 2).

**Table 2.** Estimates for the effects of distance, disability (disability vs. non-disabled) and participant sex, as well as pathogen disgust, germ aversion, perceived infectability and concern about contracting COVID-19 on comfort and attractiveness ratings

	Comfort ratings					Attractiveness ratings				
	Estimate	*SE*	*df*	*t*	*p*	Estimate	*SE*	*df*	*t*	*p*
(Intercept)	4.49	0.17	164.6	27.13	<.001***	4.86	0.19	134.4	25.68	<.001***
Distance	0.01	0.01	7442	19.15	<.001***	0.01	0.01	7442	2.19	.028*
Disability (non-disabled)	0.07	0.09	7442	0.82	.411	0.40	0.06	7442	6.59	<.001***
Sex (male)	0.15	0.24	164.6	0.63	.527	0.08	0.27	134.4	0.30	.765
Distance × disability (non-disabled)	0.01	0.01	7442	1.87	.061	0.01	0.01	7442	0.85	.396
Distance × sex (male)	−0.01	0.01	7442	−3.31	<.001***	−0.01	0.01	7442	−1.72	.086
Disability (non-disabled) × sex (male)	0.15	0.13	7442	1.16	.246	0.08	0.09	7442	0.89	.371
Distance × disability (non-disabled) × sex (male)	−0.01	0.01	7442	−0.96	.338	−0.01	0.01	7442	−0.22	.827
Pathogen disgust	0.01	0.1	122	0.179	.858	0.03	0.13	122	0.29	.772
Germ aversion	−0.02	0.12	122	−0.17	.864	−0.09	0.15	122	−0.68	.496
Perceived infectability	0.03	0.09	122	0.338	.735	0.02	0.12	122	0.21	.832
Concern about contracting COVID − 19	0.03	0.06	122	0.632	.528	0.10	0.08	122	1.33	.186

*Note:*

* *p* < .05, ***p < .001.

Another linear mixed model was conducted to investigate the effect of disability, distance, and participants’ sex on the attractiveness ratings, with participants as a random factor. Results showed that there was a very small but positive association between distance and attractiveness ratings, *β* = 0.01, *SE* = 0.01, *df* = 7442, *t* = 2.19, *p* = .028. The main effect of disability was also significant, *β* = 0.40, *SE* = 0.06, *df* = 7442, *t* = 6.59, *p* < .001 (see Table 2 for details of model). Post-hoc analysis showed that participants rated non-disabled stimuli (*M* = 4.94, *SEM* = 0.13, 95% CI [4.68, 5.20]) more attractive than disabled stimuli (*M* = 5.41, *SEM* = 0.13, 95% CI [5.15, 5.67], *p* < .001; Fig. 5B). None of the covariates were associated with attractiveness ratings (Table 2).

#### Correlation analysis

To further explore the association of comfort ratings and the measures of pathogen disgust, perceived vulnerability to disease and concern about contracting COVID-19, an average of distance preference using only the rating of disabled stimuli was calculated for each participant from all 31 stimuli. No significant correlation was found between comfort ratings from disabled stimuli with perceived infectability (*r*(120) = .04, *p* = .601), germ aversion (*r*(120) = –.04, *p* = .642), pathogen disgust (*r*(120) = –.08, *p* = .328) and concern about contracting COVID-19 (*r*(120) = .14, *p* = .119). Similarly, the attractiveness ratings were not associated with perceived infectability (*r*(120) = .04, *p* = .626), germ aversion (*r*(120) = –.01, *p* = .996) and pathogen disgust (*r*(120) = –.03, *p* = .766). However, attractiveness rating and concern about contracting COVID-19 were associated, (*r*(120) = .20, *p* = .025), albeit in an unforeseen direction: an increase with concern of contracting COVID-19 in public was associated with an increase in attractiveness ratings of the images of a disabled person.

### 3.3. Discussion

The results of the second study showed that comfort for a social interaction did not vary significantly as a function of disability; however, participants rated non-disabled stimuli more attractive than disabled ones. The results suggest that although disability clearly contributed to the attractiveness ratings, it did not influence comfort ratings for a social interaction, which is inconsistent with the predictions of the behavioural immune system (Park et al., 2003). Additionally, none of the variables of perceived infectability, germ aversion, pathogen disgust, and concern about contracting COVID-19 were associated with comfort ratings for a social interaction. This also fails to align with the proposal of the behavioural immune system. A similar pattern of results was found for ratings of attractiveness, save for its positive association with concern about contracting COVID-19, the direction of which is opposite to what the behavioural immune system would predict. Collectively, these findings are in line with and extend the previous report by Pazhoohi et al. (2022) that failed to find an association between attractiveness ratings of disabled individuals as romantic partners and perceived infectability, germ aversion, pathogen disgust and sexual disgust.

Finally, the results showed in general that men compared to women were less comfortable with a social interaction, and while this comfort rating increased with distance for both sexes, the effect of distance was weaker for men than women. Although women in general might report higher social anxiety than men (Caballo, Salazar, Irurtia, Arias, & Hofmann, 2014; Jalnapurkar, Allen, & Pigott, 2018), the sex difference in positive attitude by women compared to men towards disability is in line with recent reports, where women but not men have reported a preference for a disabled opposite sex individual (Pazhoohi et al., 2021, 2022).

## 4. Study 3

Study 1 found that wearing prosthetics is comparable to non-disabled stimuli, both in terms of comfort and attractiveness ratings. Study 2 showed that comfort ratings similarly did not differ as a function of disability (missing a limb), although participants rated non-disabled stimuli more attractive than disabled ones. These two studies, however, were limited in the fact that they used 2D stimuli on screen. In contrast to a non-immersive 2D medium, an immersive 3D environment offers greater realism and ecological validity (Parsons, 2015; Snow & Culham, 2021). Study 3 addressed this limitation by employing VR and examining again the Park et al. (2003) prediction that physical disability will be associated with larger comfort distances. Moreover, we manipulated and measured the sexes in the potential social interactions, addressing another limitation of Studies 1 and 2 (use of only female models). Finally, instead of comfort ratings (Studies 1 and 2), the third study focused on directly quantifying the comfort distance for a social interaction.

### 4.1. Method

#### Participants

A G*Power analysis for a 2 × 2 × 3 mixed-effects design indicated that 28 participants would be sufficient to detect a moderate effect size (*f* = .20, *β* = .80). However, as this study demanded significant resources to implement in terms of both physical space and programming time, we over-sampled to protect against any participant attrition (e.g. failure to arrive for testing or to follow instructions). A total of 51 individuals (33 women), aged between 18 and 35 years (*M* = 20.84, *SD* = 3.43), were recruited from the University of British Columbia and participated in this study in exchange for course credit.

#### Stimuli

**Figure 7.**
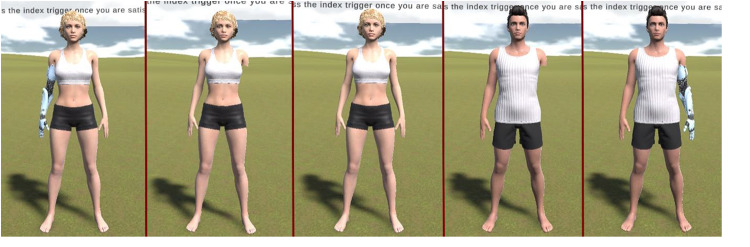
Example of female and male stimuli differing in disability in virtual reality (from left to right: disabled female with prosthetics, disabled female without prosthetics, nondisabled female, disabled male without prosthetics, and disabled male with prosthetics).

A female and a male avatar were implemented using Daz3d software (www.daz3d.com) for the non-disabled condition. Four disabled stimuli were created for the disabled condition: one where the left arms of the two avatars were removed and one where the right arms were removed. Finally, for the prosthetics condition, four stimuli were created by replacing the amputated left or right limb of the avatars with prosthetics (see Fig. 7). This yielded a total of 10 stimuli, 5 stimuli for each sex (a non-disabled stimulus, two stimuli with left and right prosthetics and two stimuli with left and right arm amputation).

#### Equipment and procedure

Upon entry into the lab, participants were given a consent form as well as a general demographic questionnaire. The participants were then brought into the VR testing room, which contained an HTC Vive Pro VR headset and two controllers. The headset screen covers approximately 110 degrees of field of view with a resolution of 12,880 × 1600 pixels and a refresh rate of 90 Hz.

Once in the virtual environment, participants underwent 50 trials. In each trial, a participant was presented with a randomly selected avatar presented at a randomly selected distance of 1, 2, 3, 4 or 5 m from the observer (10 stimuli × 5 distances = 50 trials). The participants used the touchpad on the controller to move the avatar closer or further away from them until they had positioned the avatar at the nearest socially comfortable distance from themselves. The specific instruction was to ‘Move the person in front of you so that they are positioned at the nearest comfortable distance from yourself for a social interaction.’ After being satisfied and deciding on a comfortable distance, the participants confirmed their choice by pressing the trigger on the controller, and moved to the next trial in the study.

### 4.2. Results

A 2 (participant sex) × 2 (stimulus sex) × 3 (disability condition: non-disabled, disabled, and prosthetics) mixed ANOVA was performed on the social comfort distance with participant sex as a between-subjects variable, and stimulus sex and disability condition as within-subjects variables.

The main effect of disability was significant, *F*(2, 98) = 5.92, *p* = .004, η^2^ = 0.11. Post-hoc comparisons showed that participants preferred non-disabled stimuli to be closer (*M* = 1.62, *SEM* = 0.10, 95% CI [1.42, 1.82]) compared to disabled stimuli (*M* = 1.71, *SEM* = 0.10, 95% CI [1.49, 1.92], *p* = .049) and those with prosthetics (*M* = 1.72, *SEM* = 0.11, 95% CI [1.50, 1.94], *p* = .017). No difference was found in the comfort distance for disabled and prosthetic stimuli (*p* = .999). No other main effects and none of the interactions were significant (all *p*s > .113).

### 4.3. Discussion

Study 3 investigated the effect of physical disability and prosthetics on the social comfort distance in VR. Both men and women preferred non-disabled individuals to be closer than disabled individuals, selecting greater and equivalent distances for disabled male and female avatars regardless of whether they were wearing a prosthetic. This finding is in line with the proposal that physical disability and other non-contagious physical disfigurations activate a false positive disease-avoidance cognitive process, resulting in physical avoidance and other prejudicial responses towards such individuals (Murray & Schaller, 2016; Oaten et al., 2011; Park et al., 2003).

Recent findings have pointed to the lack of negative effects of physical disability on attractiveness perception (Pazhoohi et al., 2021, 2022). Pazhoohi and colleagues (2022) tested the association of individual differences in pathogen disgust and perceived vulnerability to diseases with attractiveness ratings of disabled individuals as romantic partners. The authors found no such relationship, suggesting the failure of the Park et al. (2003) hypothesis at the level of individual differences in pathogenic sensitivity and attractiveness ratings of disabled individuals (Pazhoohi et al., 2022). A similar lack of association between individual differences in pathogen disgust and comfort ratings was found in Studies 1 and 2 of the current research. The results of Study 3, however, support Park et al.’s proposal, showing that individuals preferred larger distances from disabled avatars than non-disabled ones, regardless of participant and avatar sex. This discrepancy between the data on individual differences (Studies 1 and 2 of the current research, and Pazhoohi et al., 2022) and distance preference (Study 3) might result from differences in the ecological validity of the experiments: although in Study 3 an immersive 3D environment was employed, all the other studies used 2D stimuli on the screen. In other words, it might be the case that the cognitive disease-avoidance processes in response to physical disability are activated when interacting with 3D compared to 2D stimuli, where the realism is more enhanced. This finding needs to be investigated further in the future research; for instance, will a similar finding appear for perceived attractiveness when implemented in VR? Furthermore, it might be possible that the discrepancy in findings across studies (1 and 2 vs. 3) is due to how the instructions were worded, as well as differences in the nature of the measurements (comfort ratings vs. comfort distance). Another plausible explanation for the differences between Studies 1 and 2 and that of Study 3 might be the distinction between explicit and implicit attitudes. Participants may be reluctant to openly express discomfort with disabilities in survey responses, potentially due to social desirability biases (Pazhoohi et al., 2021). In contrast, the more subtle, implicit measure of comfort distance might more accurately reflect their true feelings, as it is less influenced by the need to conform to socially acceptable responses. Nonetheless, the results of the Study 3 using VR provides support for the ‘false positive’ negative responses against individuals with physical disabilities suggested by the behavioural immune system (Murray & Schaller, 2016; Oaten et al., 2011; Park et al., 2003).

One limitation of Studies 1 and 2 is that a single avatar was used, making it difficult to determine whether participants’ responses were influenced by the specific characteristics of that particular avatar or by the factors being hypothesized. In other words, the current studies were limited by the lack of variability in the models used as stimuli, influencing the range and applicability of our findings. In a fourth study, this limitation is addressed by employing two additional generated avatars.

## 5. Study 4

### 5.1. Method

#### Participants

A total of 201 individuals (98 men and 103 women), aged between 18 and 79 years (*M* = 37.9, *SD* = 13.4), were recruited from Prolific in the summer of 2024. A total of 97 participants (48.3%) reported being married, and 18.9% reported being not married but in a relationship. Additionally, 25.9% reported being single, and 6.9% were either widowed, divorced or separated. As for their highest educational degree, 28.9% had a high school diploma, 7.5% had a post-secondary diploma, 41.8% had an undergraduate degree and 21.8% had a postgraduate degree.

#### Measures, stimuli and procedure

**Figure 8.**
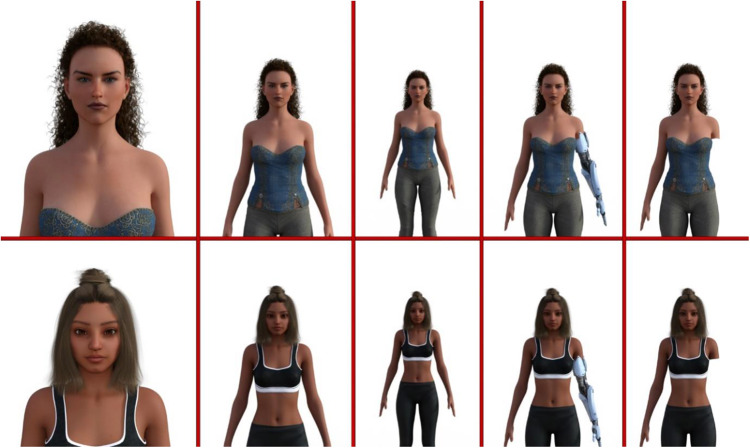
From left to right: nondisabled stimuli at distances of 100 cm, 250 cm, and 400 cm from the camera, followed by a stimulus with a prosthetic arm at 300 cm, and a disabled stimulus also at 300 cm from the camera.

Two female avatars were generated, similar to the previous 2D studies, with their distance from the camera ranging from 100 cm to 400 cm in 25 cm increments, resulting in 13 different stimuli. Additionally, two more sets of 13 images were created: one featuring an amputated arm and the other incorporating a prosthetic arm, totalling 39 stimuli corresponding to three conditions – non-disabled, disabled and prosthetic (see Fig. 8 for examples of the stimuli).

At the start of the study, participants completed a perceived vulnerability to disease scale, along with demographic questions. The stimuli sets (non-disabled, disabled and prosthetic) were then presented randomly in separate blocks. Participants were randomly assigned to one of the two avatars (half rated one avatar under all three conditions, and the other half rated the second avatar). The order of conditions (non-disabled, disabled and prosthetic) was counter-balanced across participants. As in Studies 1 and 2, participants rated each stimulus for comfort distance and attractiveness.

To assess whether participants perceived the stimuli as being at different distances or as revealing both the body and face, a manipulation check was conducted. At the conclusion of the study, participants were randomly shown three stimuli at distances of 100 cm, 250 cm and 400 cm. They were then asked, ‘How far away do you perceive the individual in the image to be from you?’ Participants indicated the perceived distance on a slider scale ranging from 100 to 400 cm.

### 5.2. Results

A linear mixed model was conducted to investigate the effect of disability, distance and participants’ sex on the comfort ratings, with participants as a random factor. Disability and participants’ sex were categorical variables and distance was continuous. Germ aversion and perceived vulnerability were added as covariates to the model. Results showed a significant main effect of disability (*F*(2, 7638) = 19.81, *p* < .001). Pairwise comparisons showed that participants indicated higher comfort ratings for non-disabled stimuli (*M* = 5.98, *SE* = 0.08), compared to disabled (*M* = 5.60, *SE* = 0.08, *p* < .001) and those with prosthetics (*M* = 5.78, *SE* = 0.08, *p* < .001). Moreover, stimuli with a prosthetic arm were rated higher for comfort than disabled stimuli (*p* < .001). No significant effect of distance, participants’ sex, germ aversion, perceived infectability or any significant interaction was found (all *p*s > .148).

Results of for the attractiveness showed a significant main effect for disability (*F*(2, 7638) = 41.20, *p* < .001); participants rated non-disabled stimuli (*M* = 5.42, *SE* = 0.10) more attractive compared to disabled (*M* = 4.39, *SE* = 0.10, *p* < .001) and those with prosthetics (*M* = 5.10, *SE* = 0.10, *p* < .001). Stimuli with a prosthetic arm were rated more attractive than disabled stimuli (*p* < .001). No significant effect of distance, participants’ sex, germ aversion, perceived infectability or any significant interaction was found (all *p*s > .053).

#### Manipulation check

The results indicated a significant effect for the manipulation check, *F*(2, 398) = 291.46, *p* < .001. Participants perceived the stimuli at distances of 100 cm, 250 cm and 400 cm as significantly different from each other (all *p*s < .001). The mean perceived distances were as follows: 100 cm stimulus (*M* = 53.28, *SE* = 4.72, 95% CI [43.99, 62.58]), 250 cm stimulus (*M* = 120.99, *SE* = 5.86, 95% CI [109.44, 132.54]) and 400 cm stimulus (*M* = 187.36, *SE* = 7.84, 95% CI [171.89, 202.83]).

### 5.3. Discussion

The results of Study 4 extended the findings of the previous studies by employing multiple avatars, addressing a limitation noted in Studies 1 and 2 where only a single avatar was used. Specifically, participants provided higher comfort and attractiveness ratings for non-disabled individuals than both disabled individuals and those with prosthetics. Additionally, stimuli with a prosthetic arm were rated higher in both comfort and attractiveness than disabled stimuli, suggesting a nuanced perception of disability where prosthetics may mitigate some of the negative biases associated with physical disabilities. The significant effect of disability on both comfort and attractiveness ratings aligns with findings from Studies 2 and 3, where non-disabled stimuli were rated more positively than both disabled stimuli and those with prosthetics.

The consistent finding across Studies 3 and 4 that disability influences comfort distance and comfort ratings reinforces the notion that physical disability triggers a negative response, likely rooted in disease-avoidance mechanisms as proposed by the behavioural immune system (Park et al., 2003). However, the lack of association between comfort ratings and individual differences in germ aversion or perceived infectability, which was also observed in Studies 1 and 2, continues to challenge this theoretical framework. Overall, these results indicate that although physical disability influences comfort distance in social interactions, individual differences in pathogen disgust and vulnerability to disease do not appear to affect how comfortable people feel in these interactions.

## 6. General Discussion

The current research investigated comfort for a social interaction, and perceived attractiveness, as a function of distance when individuals viewed non-disabled and prosthetic images of a female avatar (Study 1), as well as non-disabled and disabled images of a female avatar (Study 2). Study 3 extended Studies 1 and 2 by employing VR to providing more ecological validity, as well as including a male avatar. Study 4 further extended Studies 1 and 2 by introducing additional avatars. We also measured individual differences in pathogen disgust, perceived vulnerability to disease and concern of contracting COVID-19. The current research tests for the first time a proposal based on behavioural immune system that physical disability and other physical disfiguring conditions can lead to negative cognitive and behavioural responses (i.e. comfort distance), thereby inducing prejudicial attitudes and avoidance behaviours towards individuals with physical disabilities (Murray & Schaller, 2016; Park et al., 2003).

The results showed that the comfort ratings for a social interaction did not vary significantly as a function of disability in Studies 1 and 2. Participants did not rate comfort differently from non-disabled stimuli, whether it was for the prosthetics (Study 1) or physical disability (Study 2). However, participants rated non-disabled stimuli more attractive than disabled ones (Study 2). Using a more varied set of avatars, the results of Study 4 revealed that disability significantly impacted both attractiveness and comfort ratings in social interactions. Participants rated non-disabled avatars higher in comfort and attractiveness compared to both disabled and prosthetic avatars. Additionally, stimuli with prosthetics were rated higher in attractiveness and comfort than those with disabilities. However, none of the variables of perceived infectability, germ aversion, pathogen disgust and concern about contracting COVID-19 were associated with comfort ratings for a social interaction in Studies 1, 2 and 4. This pattern of results does not support the predictions by the behavioural immune system in which individual differences in pathogen disgust and vulnerability to disease are associated with prejudicial attitudes and avoidance behaviours towards individuals with physical disabilities (Park et al., 2003). In Study 3 where using VR we explored the hypothesis that whether social comfort distance is influenced by physical disability and if wearing a prosthetic reduces that distance, results showed that individuals, regardless of sex, preferred to stand closer to non-disabled male and female avatars than disabled ones. This preference for a greater distance from disabled individuals was not reduced when prosthetics were worn. The findings from Studies 3 and 4 consistently demonstrate that physical disability affects both comfort distance and comfort ratings, supporting the idea that such disabilities may elicit negative reactions, potentially driven by disease-avoidance mechanisms as suggested by the behavioural immune system (Park et al., 2003). This theory posits that humans have evolved cognitive processes designed to avoid potential sources of disease, which can manifest as discomfort or avoidance behaviours toward individuals perceived as physically different or impaired. However, the absence of a significant relationship between comfort ratings and individual differences in germ aversion or perceived infectability, as observed in Studies 1, 2 and 4, challenges this theoretical perspective. If the behavioural immune system were strongly at play, we would expect individuals with higher sensitivity to pathogens (as measured by germ aversion and perceived infectability) to report lower comfort levels in social interactions involving disabled individuals. Yet, this expected pattern did not emerge across multiple studies. In other words, although disability clearly influences how people judge comfort and appropriate social distance, individual differences in pathogen-related concerns do not seem to modulate these judgements. This might suggest that the discomfort associated with interacting with disabled individuals may stem from factors other than disease avoidance, such as social stigma or preconceived notions about disability, rather than an innate response to potential disease threats.

One limitation worth noting in all four studies is the use of video-game-like characters as stimuli. Using manipulated photographs of real people might more effectively evoke genuine reactions to disabilities, as they could provide a more realistic context for assessing people’s responses in real-world scenarios. This approach could potentially offer a deeper insight into the behavioural immune system’s impact on social perceptions of disabilities. Furthermore, in Studies 1, 2 and 4 the distance may be confounded with the amount of information available; as distance increases, more of the avatar is visible, revealing more details such as waist and hip measurements, which could affect perceptions of attractiveness. This limitation was addressed in the third study, where participants had the opportunity to evaluate different aspects of the models for a more comprehensive assessment. Moreover, the manipulation check conducted in Study 4 showed that participants perceived the avatars as being at different distances. Although attractiveness was not the primary focus of our research – rather, comfort distance was – the additional visual information available in the first two studies as well Study 4 might have influenced participant decisions. Therefore, future research could include a manipulation check allowing for open-ended responses to clarify whether distance or visual factors influence perception.

In conclusion, across four studies the current research provided insights into the relationship between physical disability, social comfort and perceived attractiveness. In Studies 1 and 2, comfort ratings for social interactions were not significantly affected by disability. Moreover, perceived infectability, germ aversion, pathogen disgust and COVID-19 concern did not correlate with comfort ratings, contradicting the predictions of the behavioural immune system. In Study 3 in a VR environment, participants preferred to stand closer to non-disabled avatars compared to disabled avatars, with and without the presence of prosthetics. This outcome was replicated in a fourth study where a more varied set of 2D stimuli was used. Altogether, this research showed that physical disability affects comfort distance and ratings, suggesting negative reactions linked to disease-avoidance mechanisms. However, the lack of a relationship between comfort ratings and individual differences in pathogen disgust suggests that this discomfort may stem more from social stigma or preconceived notions than from an innate disease-avoidance response, challenging the behavioural immune system proposal.

## Supporting information

Pazhoohi et al. supplementary material Pazhoohi et al. supplementary material

## Supplementary material

The supplementary material for this article can be found at https://doi.org/10.1017/ehs.2025.5.

## Research transparency and reproducibility interest

Data are available in the supplementary material.
